# Supplementary material for: Sex Differences in Gut Microbiota and Their Relation to Arterial Stiffness (MIVAS Study)
Source: Nutrients. 2024 Dec 27;17(1):53. doi: 10.3390/nu17010053 (PMC11723250; doi:10.3390/nu17010053)
Supplement: Supplementary file 1 [file nutrients-17-00053-s001.zip › nutrients-3366399-supplementary.pdf]

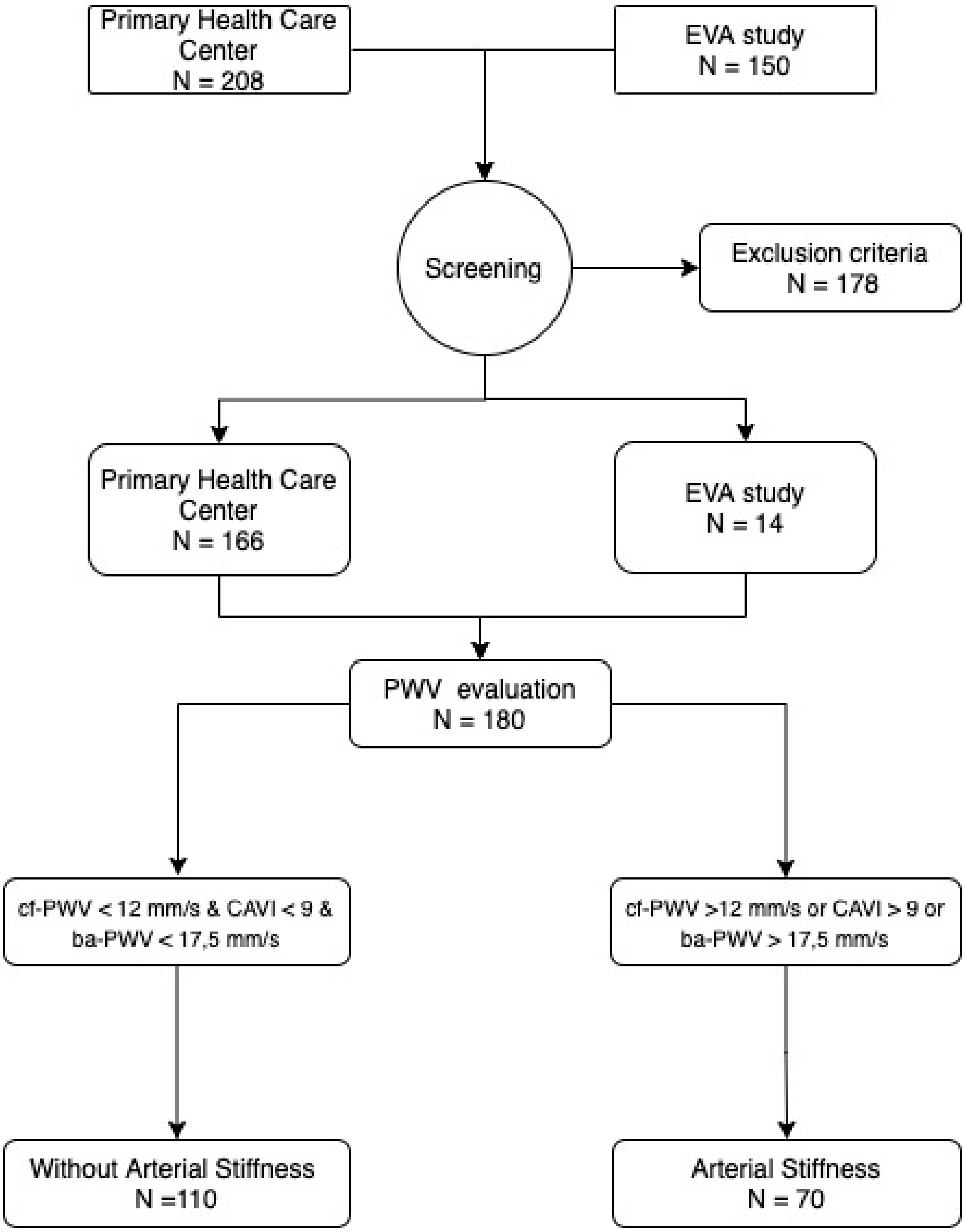

Figure S1: Study flow chart. cf-PWV carotid-femoral PWV; CAVI, Cardio-Ankle Vascular Index; ba-PWV, brachial-ankle PWV; PWV, pulse wave velocity.

Table S1. Vascular function parameters

	Total (180)		Women (n= 122; 68%)		Men (58; 32%)		Women vs Men
	Mean/N	SD/%	Mean/n	SD/%	Mean/n	SD/%	p-value
cf-PWV mean	10.6	2.3	10.4	2.1	11.2	2.6	0.04
CAVI_mean	8.4	1.0	8.3	1.0	8.5	1.1	0.12
ba-PWV mean m/sec	14.2	2.5	14.2	2.4	14.1	2.6	0.74
cf-PWV > 12 m/s (n/%)	34	18.8	20	16.4%	14	24.1%	0.23
CAVI> 9 (n/%)	51	28.3	31	25.4%	20	34.5	0.34
ba-PWV > 17.5 m/sec (n/%)	20	11.1	11	9.0%	9	15.5	0.20
Arterial Stiffness Group (n/%)	70	38.8	45	36.8	25	43.1	0.52

cf-PWV: carotid femoral pulse wave velocity; CAVI: Cardio ankle vascular index; baPWV: brachial ankle pulse wave velocity; SD: Standard Deviation

**Table S2. Genus differential expression in women vs men**				
Genus	logFC	logCPM	PValue	FDR
Blautia	0.96	16.18	<0.01	<0.01
Ruminococcus	0.11	14.71	0.64	0.73
Subdoligranulum	0.12	14.38	0.48	0.60
Bacteroides	-0.45	16.56	<0.01	0.01
Roseburia	1.74	15.27	<0.01	<0.01
Dorea	2.08	14.73	<0.01	<0.01
Ruminococcaceae UCG002	0.11	14.71	0.64	0.73
Faecalibacterium	-0.36	16.39	<0.01	<0.01
Bifidobacterium	0.49	14.65	0.04	0.079
Agathobacter	1.52	14.49	<0.01	<0.01
Christensenellaceae_R-7_group	-0.68	13.95	0.01	0.03

Genus differential expression in women vs men. *Dorea, Roseburia y Agathobacter* are more abundant in women. **Log FC:** log fold change. **LogCPM:** log count per million**. FDR:** False Discovery Rate.

**Table S3. Genus differential expression in women vs men in arterial stiffness subjects**				
Genus	logFC	logCPM	PValue	FDR
Blautia	2.30	16.11	<0.01	<0.01
Ruminococcus	0.35	14.84	0.33	0.54
Subdoligranulum	0.08	14.28	0.76	0.85
Bacteroides	0.60	16.59	0.02	0.05
Roseburia	2.29	15.36	<0.01	<0.01
Dorea	3.27	14.68	<0.01	<0.01
Ruminococcaceae UG 002	0.27	14.32	0.39	0.61
Faecalibacterium	-0.69	16.40	<0.01	0.01
Bifidobacterium	0.81	14.59	0.03	0.07
Agathobacter	1.98	14.65	<0.01	<0.01

Genus differential expression in women vs men. *Dorea, Roseburia, Blautia and Agathobacter* are more abundant in women, **Log FC:** log fold change. **LogCPM:** log count per million**. FDR:** False Discovery Rate.

**Table S4. Logistic regression relating microbiome abundance (genus) with arterial stiffness (all subjects)**									
	Model 1			Model 2			Model 3		
	OR	IC	p-value	OR	IC	p-value	OR	IC	p-value
Blautia	0.93	(0.67-1.27)	0.63	0.87	(0.6-1.27)	0.48	0.88	(0.61-1.29)	0.52
Ruminococcus	0.98	(0.71-1.36)	0.92	0.96	(0.66-1.38)	0.81	0.95	(0.66-1.37)	0.8
Ruminococcaceae UCG002	1.12	(0.78-1.62)	0.54	1.08	(0.72-1.62)	0.72	1.07	(0.72-1.61)	0.73
Anaerostipes	1.43	(1.02-2)	0.04	1.44	(1.01-2.06)	0.05	1.46	(1.02-2.1)	0.04
Bacteroides	0.76	(0.54-1.06)	0.11	0.82	(0.57-1.17)	0.27	0.82	(0.57-1.19)	0.3
Roseburia	0.69	(0.48-0.98)	0.04	0.68	(0.47-1)	0.05	0.69	(0.47-1.01)	0.06
Dorea	0.95	(0.67-1.33)	0.76	0.94	(0.64-1.38)	0.76	0.96	(0.65-1.42)	0.84
Christensenellaceae _R7_group	0..97	(0.69-1.38)	0.88	0.85	(0.59-1.22)	0.38	0.85	(0.59-1.23)	0.39
Faecalibacterium	0.8	(0.56-1.14)	0.21	0.82	(0.57-1.18)	0.29	0.81	(0.56-1.17)	0.26
Bifidobacterium	1.15	(0.82-1.6)	0.42	1.02	(0.7-1.48)	0.91	1.02	(0.7-1.48)	0.92
Agathobacter	0.7	(0.5-0.99)	0.04	0.73	(0.5-1.07)	0.11	0.73	(0.5-1.06)	0.1
Subdoligranulum	1.25	(0.89-1.76)	0.2	1.16	(0.79-1.69)	0.45	1.14	(0.78-1.66)	0.51

Logistic regression of microbiome abundance (independent variable) and arterial stiffness (dependent variable). Model 1: adjusted for sex and age. Model 2: adjusted for model 1 and body mass index, hemoglobin A1c, total cholesterol, HDL cholesterol, LDL cholesterol, and systolic blood pressure. Model 3: adjusted for model 2 and mediterranean diet score .* p-value <0.05

Table S5. Logistic regression relating microbiome abundance (genus) with arterial stiffness by sex

	Model 1			Model 2			Model 3		
**Men**									
	OR	IC	p-value	OR	IC	p-value	OR	IC	p-value
Blautia	0.77	(0.41-1.43)	0.4	0,58	(0.26-1.3)	0.19	0.58	(0.25-1.33)	0.2
Ruminococcus	0.81	(0.42-1.55)	0.52	0.67	(0.3-1.48)	0.33	0.67	(0.3-1.49)	0.33
Ruminococcaceae UCG002	1.02	(0.52-2.01)	0.96	0.99	(0.42-2.35)	0.98	0.98	(0.41-2.34)	0.96
Anaerostipes	1.18	(0.6-2.34)	0.63	1.85	(0.83-4.09)	0.14	1.82	(0.82-4.04)	0.15
Bacteroides	0.8	(0.41-1.55)	0.5	0.73	(0.32-1.67)	0.47	0.73	(0.32-1.67)	0.46
Roseburia	0.62	(0.3-1.32)	0.22	0.23	(0.07-0.79)	0.02	0.23	(0.07-0.81)	0.03
Dorea	0.75	(0.4-1.42)	0.38	0.53	(0.26-1.09)	0.09	0.53	(0.26-1.1)	0.1
Christensenellaceae_ R7_group	1.33	(0.71-2.48)	0.38	1.26	(0.59-2.66)	0.55	1.24	(0.57-2.7)	0.6
Faecalibacterium	1.1	(0.57-2.13)	0.78	1.27	(0.6-2.66)	0.54	1.26	(0.6-2.66)	0.54
Bifidobacterium	2.56	(1.3-5.07)	0.01	2.34	(1.04-5.26)	0.04	2.52	(1.03-6.13)	0.05
Agathobacter	0.74	(0.4-1.38)	0.35	0.64	(0.32-1.3)	0.22	0.65	(0.32-1.33)	0.24
Subdoligranulum	2.18	(0.99-4.8)	0.06	2.85	(1.12-7.22)	0.03	3.41	(1.2-9.64)	0.03
**Women**									
	Model 1			Model 2			Model 3		
labels	OR	IC	p-value	OR	IC	p-value	OR	IC	p-value
Blautia	0.98	(0.67-1.45)	0.94	0.9	(0.56-1.46)	0.68	0.94	(0.58-1.53)	0.8
Ruminococcus	1.08	(0.73-1.59)	0.7	1.02	(0.65-1.59)	0.95	1.02	(0.65-1.59)	0.94
Ruminococcaceae UCG002	1.26	(0.79-2.01)	0.33	1.33	(0.79-2.23)	0.28	1.29	(0.77-2.16)	0.33
Anaerostipes	1.46	(0.98-2.17)	0.07	1.46	(0.95-2.26)	0.09	1.49	(0.96-2.33)	0.08
Bacteroides	0.74	(0.49-1.1)	0.14	0.73	(0.46-1.16)	0.19	0.74	(0.47-1.18)	0.21
Roseburia	0.67	(0.44-1.03)	0.07	0.68	(0.43-1.07)	0.1	0.7	(0.44-1.1)	0.12
Dorea	1.08	(0.72-1.64)	0.7	1.19	(0.71-2)	0.51	1.25	(0.74-2.12)	0.41
Christensenellaceae_ R7_group	0.96	(0.64-1.44)	0.84	0.9	(0.57-1.42)	0.66	0.88	(0.56-1.4)	0.6
Faecalibacterium	0.7	(0.46-1.08)	0.11	0.73	(0.47-1.14)	0.17	0.7	(0.45-1.09)	0.12
Bifidobacterium	0.93	(0.64-1.36)	0.71	0.92	(0.59-1.44)	0.72	0.9	(0.57-1.42)	0.66
Agathobacter	0.69	(0.46-1.01)	0.06	0.67	(0.42-1.06)	0.09	0.67	(0.42-1.06)	0.09
Subdoligranulum	1.1	(0.72-1.67)	0.67	0.91	(0.54-1.53)	0.72	0.87	(0.51-1.47)	0.59

Logistic regression of microbiome abundance (independent variable) and arterial stiffness (dependent variable) in men and women. Model 1: adjusted for age. Model 2: adjusted for model 1 and body mass index, hemoglobin A1c, total cholesterol, HDL cholesterol, LDL cholesterol, and systolic blood pressure. Model 3: adjusted for model 2 and mediterranean diet score.* p-value <0.05.
